# Diabetes Distress Among Type 1 Diabetic Adolescents in a Tertiary Care Hospital in Pakistan

**DOI:** 10.7759/cureus.32392

**Published:** 2022-12-11

**Authors:** Zaina Jabeen, Amena M Baig, Khadija I Khawaja, Sumayya Shabbir, Zubana Afzal

**Affiliations:** 1 Department of Endocrinology and Metabolism, Services Hospital Lahore, Lahore, PAK; 2 Department of Endocrinology and Metabolism, Services Institute of Medical Sciences, Lahore, PAK; 3 Department of Internal Medicine, Mayo Hospital, Lahore, PAK; 4 Department of Applied Psychology, Punjab University, Lahore, PAK

**Keywords:** diabetes and mental health, adolescents with diabetes, diabetes-distress, diabetes self-management, type i diabetes mellitus

## Abstract

Background and Aim: Diabetes distress, a term used to describe negative emotions associated with diabetes, is the key factor responsible for the elevated risk of psychological burden and compromised self-management. The aim of this study is to determine the prevalence of diabetes-related distress among adolescent patients with type 1 diabetes (T1D) and to ascertain various factors associated with it.

Methodology: In this cross-sectional study, 117 T1D patients with age 12-20 years visiting a diabetic clinic in the Department of Endocrinology and Metabolism, Services Hospital Lahore from February 2022 to August 2022 were enrolled. The patient’s demographic and clinical details were noted in a pre-designed proforma. T1D distress scale (T1DDS) was utilized as the tool for measuring diabetes distress and distress was classified as severe, moderate, and no/little distress.

Results: Of the total 117 T1D patients, 34.2% (n=40) had diabetes-related distress, out of which 31.6% had moderate and 2.6% had severe distress. The average total distress score was 1.73 ± 0.52 and higher mean scores were of powerlessness, negative social perception, and eating distress. Distress was higher among females, in those with the onset of diabetes in teens rather than in childhood. There is a significant impact of glycated hemoglobin (HbA1c) on the severity of diabetes distress as demonstrated by Pearson’s correlation (r=.570, n= 117, p = <.001)

Conclusion: The present study highlights the association of diabetes distress in adolescents with various factors, most significantly poor glycemic control, and therefore emphasizes the need for developing psychological interventional strategies in routine diabetes care to improve the mental well-being and self-management of diabetic patients.

## Introduction

Diabetes is the most prevalent non-communicable disease affecting more than 420 million population worldwide and is estimated to affect up to 640 million by 2040 [1]. Type 1 diabetes (T1D) is the commonest form of diabetes occurring during childhood and adolescence, responsible for up to 80% of newly diagnosed diabetes in patients ≤19 years of age [2,3]. T1D is a lifelong disease requiring insulin administration for survival. Due to its chronic nature along with associated incessant daily lifestyle changes, need for frequent self-blood glucose monitoring, daily insulin administration, fear of complications and hypoglycemia, and need for regular healthcare visits, T1D is associated with significant psychological morbidity and mental health problems [4].

Diabetes distress (DD), a distinct entity from depression, refers to the negative emotional impact of daily life with diabetes and includes feelings of frustration, anger, guilt, fear, powerlessness, and lack of motivation. The emotional burden associated with diabetes distress causes impediments in diabetes self-management, referred to as diabetes burnout [5]. Adolescents with T1D, in addition to going through the physical and psychological changes of adolescence, suffer from added distress having to deal with the daily continuing demands of diabetes [6]. Previous studies have shown a higher prevalence of DD in adolescents with T1D as compared to adults and this is also linked to reduced self-care, poor self-management, and suboptimal diabetes control [7].

Multiple validated scales exist to assess diabetes distress, but T1D Distress Scale (T1-DDS), is created specifically to assess diabetes distress in the T1D population [8-13]. The 28-item based T1-DDS scale consists of seven further subscales: powerlessness, management distress, hypoglycemia, negative social perceptions, eating distress, physician distress, and friend/family distress.

There are few local studies conducted on the prevalence of diabetes distress in type 2 diabetes [14] but there is no published data on diabetes distress in adolescents with T1D in Pakistan. Thus, this present study aimed to assess the prevalence of DD, identify various sources of DD among demographic and clinical characteristics, and determine its relationship with various factors including glycemic control among T1D adolescents in a tertiary care hospital of a developing country.

## Materials and methods

This cross-sectional study was conducted on adolescents with T1D attending a diabetes clinic in the Department of Endocrinology and Metabolism, Services Hospital Lahore, Pakistan, over a period of seven months from February 2022 to August 2022. This study was done in collaboration with a health psychologist from Punjab University, Lahore, Pakistan (ZA). Prior approval of the study protocol was sought from the Institutional Review Board of the Services Institute of Medical Sciences (Ref No. IRB/2022/1022/SIMS).

The sample size was calculated keeping prevalence at 7.2% based on estimates in previous studies [15], keeping a 95% confidence interval, 5% absolute error, and assuming a dropout rate of 10%.

Using convenient sampling, 117 patients with T1D were included in this study. Inclusion criteria included: (1) diagnosed patients of T1D in the age group of 12-20 years, (2) diabetes duration of more than six months, (3) agreeing to participate voluntarily, and (4) completing the questionnaire. Exclusion criteria included a history of any psychological illness or intake of psychotropic medication, any developmental disorder, any acute or serious illness requiring hospitalization at the time of the interview, or a duration of diabetes less than six months. Patients who had any significant life events such as the death of a loved one or any personal or financial loss within the last six months were also excluded.

Informed consent was taken from all the parents in written form and assent was sought from diabetic adolescents providing them with age-appropriate information. After taking consent, the patient’s socio-demographic and clinical details were noted in a pre-designed proforma. Clinical details included duration and age of onset of diabetes, presence of complications of diabetes, frequency and severity of hypoglycemic episodes, anthropometric measures (eg BMI), and biochemical profile (fasting blood glucose and glycated hemoglobin (HbA1c)).

After recording socio-demographic details, data pertaining to DD was recorded by the team, which included a health psychologist as well. DD score was measured using T1DDS, which is a validated 28-items scale designed specifically for T1D. A mean distress score ≤2 indicated no or little distress, 2-2.9 indicated moderate distress, and ≥3 signified severe distress. The diabetes distress score was further categorized into seven subscales as described.

Data were analyzed using IBM SPSS Statistics for Windows, Version 29.0 (Released 2022; IBM Corp., Armonk, New York, United States). Quantitative data were described as mean and standard deviation and categorical as frequency and percentages. Independent sample t-tests were used to compare distress scores between two groups and one-way ANOVA was used to compare more than two groups. Chi-Square was used to correlate distress categories (no/moderate/severe distress) with gender, duration, and age of onset of diabetes. The correlation between HbA1c and DD score was analyzed using Pearson’s correlation. Various factors influencing distress were analyzed using multiple regression. A p-value of less than 0.05 was considered statistically significant.

## Results

There were 117 T1D participants in the study, out of which 56.4% (66) were males and 43.6% (51) were females. Mean age of participants was 15.41 ± 2.48, mean age of onset of diabetes was 9.8 ± 4.76 (years), mean duration of diabetes was 5.6 ± 4.68 (yrs), mean BMI was 18.5 ± 3.2, and mean HbA1c (%) was 9 ± 1.67. Demographic and clinical characteristics are shown in Table 1.

**Table 1:** Clinical and demographic characteristics of participants For continuous variables, values are given as mean and standard deviation (SD) and for categorical variables, values are given in number (N) with percentages (%) T1D: type 1 diabetes

Characteristics		Mean / N	SD / %	Characteristics		Mean / N	SD / %
Gender	Males	66	56.4	Complications of diabetes	Present	9	7.7
	Females	51	43.6		Absent	108	92.3
Age (years)		15.41	2.48	Other auto-immune conditions	Celiac disease	6	5.1
BMI (kg/m^2^)		18.5	3.2		Thyroid dysfunction	6	5.1
Age of onset of diabetes (years)		9.8	4.76		Other	105	89
Duration of diabetes (years)		5.6	4.68	T1D-related ER visits/ admissions during last six months	0	66	56.4
HbA1c (%)		9	1.67		1-2	42	35.9
Monthly income	< 15000	65	55.5		≥4	9	7.9
	15000-30000	43	36.7	Number of hypoglycemic episodes (during last one month)	0	62	53.8
	30000-50000	7	5.9		1-3	43	35.9
	>50000	2	1.7		≥4	11	10.3

Of the 117 patients, 34.2% (n=40) had DD, out of which 31.6% (n=37) had moderate and 2.6% (n=3) had severe distress. The mean total distress score was 1.73 ± 0.52 and mean scores were higher in subscales of powerlessness, eating distress, and negative social perception. Mean distress scores for the seven subscales are shown in Table 2.

**Table 2:** Mean distress scores for subgroups

Subscale	Score
Powerlessness	2.01 ± 0.68
Management distress	1.78 ± 0.75
Hypoglycemia distress	1.32 ± 0.50
Negative social perception	1.88 ± 0.67
Eating distress	1.92 ± 0.81
Physician distress	1.62 ± 0.56
Family/friends'distress	1.60 ± 0.57

The Association of demographic and clinical characteristics of participants with mean distress score is shown in Table 3.

**Table 3:** Association of demographic and clinical characteristics of participants with mean diabetes distress score T1D: type 1 diabetes

	Sub-groups	Mean total distress score	p-value
Age	12-15	1.74	.805
	16-18	1.74	
	19-20	1.50	
Gender	Females	1.95	< .001
	Males	1.56	
Age at onset of diabetes (years)	1-5	1.69	.003
	6-10	1.64	
	11-15	1.67	
	16-20	2.20	
Complications of diabetes (e.g., neuropathy)	Present	2.21	.005
	Absent	1.69	
Other associated autoimmune conditions	Celiac	1.65	.551
	Autoimmune thyroid disease		
	None	1.74	
Primary caretaker	Self	1.73	.789
	Parent/other	1.76	
Hypoglycemic episodes during last one month	0	1.68	.625
	1-3	1.78	
	≥4	1.90	
T1D-related ER visits/ admissions during last six months	0	1.69	.247
	1-2	1.75	
	≥3	2.00	

**Figure 1:**
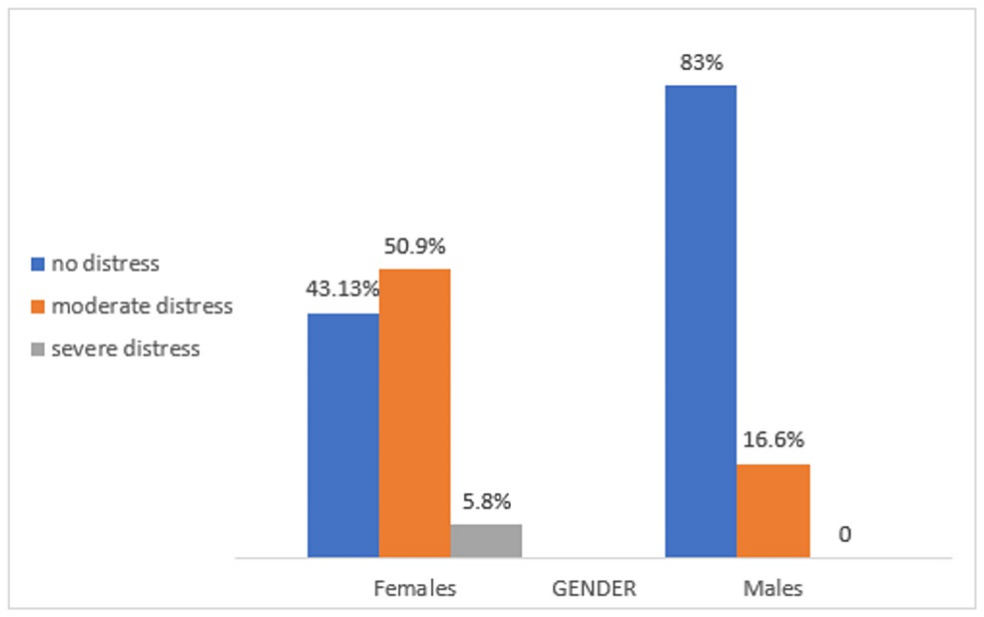
Percentage of males and females with normal, moderate, and severe distress This association is statistically significant, x2 (5) =21.65, p = .001

Among a total of 51 female participants, 26 (51%) had moderate and three (5.8%) had severe distress. Among 66 males, 11 (16.6%) had moderate distress and none had severe distress. The distribution of DD in males and females is shown in Figure 1.

**Figure 2:**
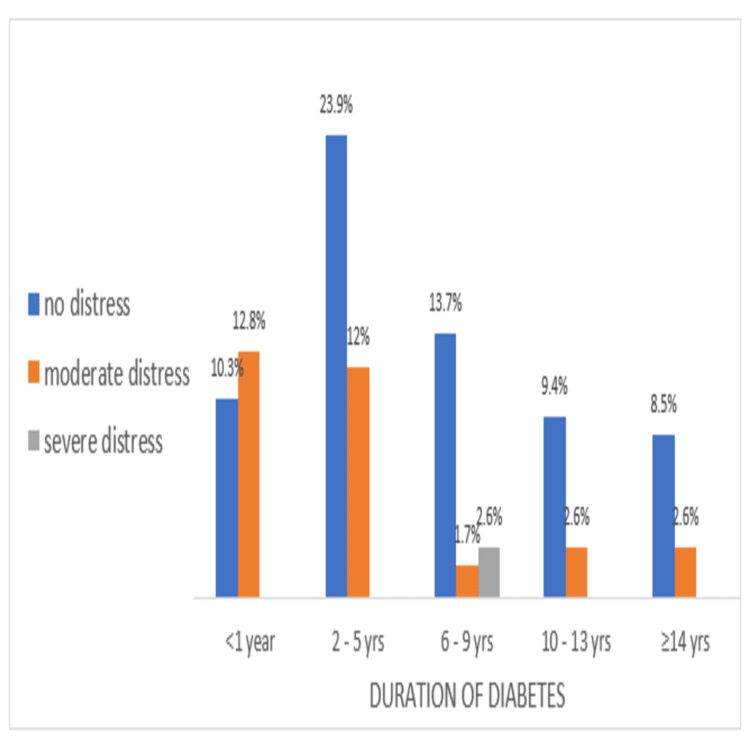
Percentage of participants with normal, moderate, and severe distress according to duration of diabetes (≤1 year, 2-5 years, 6-9 years, 10-13 years, ≥14 years) The association is statistically significant, x2 (8) =25.45, p = .001

**Figure 3:**
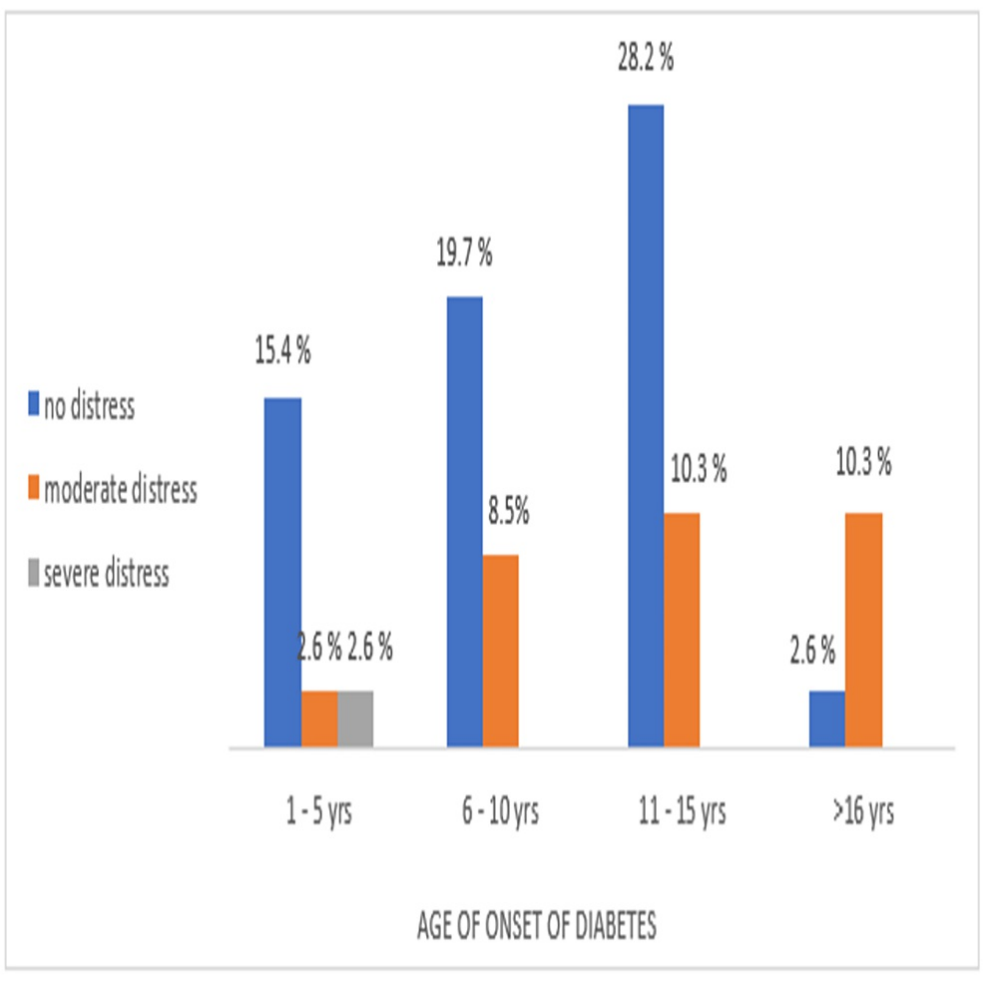
Percentage of participants with normal, moderate, and severe distress according to age of onset of diabetes (1-5 years, 6-10 years, 11-15 years, ≥16 years) The association is statistically significant, x2 (6) =31.42, p = .001

The prevalence of normal, moderate, and severe DD among the participants according to the duration of diabetes and age of onset is shown in Figures 2, 3, respectively.

Pearson’s correlation demonstrated a significant correlation between HbA1c and total distress score (r=.570, n= 117, p = <.001) showing an association of higher distress level with poor glycemic control.

A multiple regression analysis was done to predict total distress score from age, gender, recent HbA1c, age at onset, and presence of complications. The overall regression was statistically significant (F (6,110) =12.387, p <.001, R2=.403). It was found that gender (p= .002, 95%CI: .101-423) and HbA1c (p <.001, 95%CI: .099-.203) significantly altered total distress score.

## Discussion

This study was done to evaluate diabetes-related distress in young adolescents with T1D and to ascertain various underlying factors. Our study showed that 34.6% of adolescents with T1D had distress, which is higher than a few previous studies in similar age groups, which had shown the prevalence of DD to be around 20-30% [16,17]. This could be explained by the fact that the majority of patients in our study belonged to the lower-income class. A similar effect of socioeconomic class on the severity of DD has been observed in a previous study showing higher distress among patients from lower-income groups [16].

Our study showed that among adolescents, girls were more susceptible to distress. Gender differences in emotional stress responses could be the possible explanation for such differences and this gender difference has been shown in numerous previous studies as well [18,19].

 Moreover, patients with higher distress had significantly higher HbA1c and poor glycemic control. It is unclear which is cause or effect; however, the presence of distress is likely to impair glycemic control as it causes obstacles to self-management of T1D, a behavioral adjustment termed diabetes burnout. This association has been seen in previous studies [9,16,20,21].

 Our study showed that distress was higher in patients with onset of diabetes in teens as compared to those with onset in childhood. Similarly, when stratified according to the duration of diabetes, distress was higher with recent-onset diabetes than those with prolonged duration. The possible explanation for this might include pubertal physiological changes, developmental behavior, lack of diabetes education, sense of denial towards diagnosis earlier in the illness [18]. This also emphasizes the potential role of distress screening and psychological interventions beginning earlier after diagnosis of diabetes [22,23]. The relationship of distress with the duration of diabetes and distress is inconsistent in the previously published literature [24,25]. Unlike the current study, one previous study has shown a positive correlation between distress and the duration of diabetes [26].

Adolescents with T1DM need additional psychological support including screening for distress and interventional therapies in order to avoid distress during the provision of clinical care for diabetes. This might lead to better efficiency in terms of both glycemic control and mental health [27-30]. A systematic review and meta-analysis by Sturt et al. showed that following psychological interventions, there was an improvement in not only the diabetes distress but also in HbA1c levels and glycemic control [27].

The strength of this study is that it is the first study on the prevalence and associations of DD in T1D patients in Pakistan, which is often an overlooked aspect of diabetes in developing countries. It calls for the development of public health interventions to cope with DD in a limited resource setting of a developing country. This study has a few limitations. Some important variables were not assessed including social status, educational background, marital status, etc. As the majority of patients in our study belonged to a lower socioeconomic group, the results may vary from the general diabetic population. Moreover, owing to the cross-sectional nature of this study, causal inferences cannot be withdrawn, and hence the cause-effect relationship between poor glycemic control and high distress is not clear, Therefore, a longitudinal study would be needed to determine causality. Other limitations include a small sample size and being a single-center study.

## Conclusions

The present study highlights the association of DD among adolescents with various factors, most significantly poor glycemic control, emphasizing the need for regular screening for DD and developing psychological interventional strategies in routine diabetes care to improve mental health and self-management of diabetic patients.
